# Expression Profiling of Single Mammalian Cells – Small is Beautiful

**DOI:** 10.1002/1097-0061(20000930)17:3<211::AID-YEA26>3.0.CO;2-7

**Published:** 2000

**Authors:** Gerard Brady

**Affiliations:** G.38 Stopford Building School of Biological Sciences The University of Manchester Oxford Road Manchester M13 9PT UK

## Abstract

Increasingly mRNA expression patterns established using a variety of molecular technologies such as cDNA microarrays, SAGE and cDNA display are being used to identify potential regulatory genes and as a means of providing valuable insights into the biological status of the starting sample. Until recently, the application of these techniques has been limited to mRNA isolated from millions or, at very best, several thousand cells thereby restricting the study of small samples and complex tissues. To overcome this limitation a variety of amplification approaches have been developed which are capable of broadly evaluating mRNA expression patterns in single cells. This review will describe approaches that have been employed to examine global gene expression patterns either in small numbers of cells or, wherever possible, in actual isolated single cells. The first half of the review will summarize the technical aspects of methods developed for single-cell analysis and the latter half of the review will describe the areas of biological research that have benefited from single-cell expression analysis.


**Yeast**
*Yeast* 2000; **17**: 211–217.

**Review Article**

### Expression profiling of single mammalian cells – small is beautiful

Gerard Brady*
*School of Biological Sciences, G.38 Stopford Building, University of Manchester, Oxford Road, Manchester M13 9PT, UK*

**Correspondence to:*
*G. Brady, G.38 Stopford Building, School of Biological Sciences, The University of Manchester, Oxford Road, Manchester M13 9PT, UK.*
*E-mail: Ged.Brady@man.ac.uk*

Received: 13 July 2000
Accepted: 14 July 2000

### Abstract

**Increasingly mRNA expression patterns established using a variety of molecular technologies such as cDNA microarrays, SAGE and cDNA display are being used to identify potential regulatory genes and as a means of providing valuable insights into the biological status of the starting sample. Until recently, the application of these techniques has been limited to mRNA isolated from millions or, at very best, several thousand cells thereby restricting the study of small samples and complex tissues. To overcome this limitation a variety of amplification approaches have been developed which are capable of broadly evaluating mRNA expression patterns in single cells. This review will describe approaches that have been employed to examine global gene expression patterns either in small numbers of cells or, wherever possible, in actual isolated single cells. The first half of the review will summarize the technical aspects of methods developed for single-cell analysis and the latter half of the review will describe the areas of biological research that have benefited from single-cell expression analysis. Copyright © 2000 John Wiley & Sons, Ltd.**

**Keywords:** **expression profiling; single-cell; mRNA; PolyAPCR; RT–PCR; quantitative PCR**

### Introduction

Following enormous advances in the area of genomics and the complete sequencing of the human genome, the current challenge to biologists is to learn how the products of the 30 000–150 000 identified genes interact to produce the complexity exhibited by higher eukaryotes. Although an examination of mRNA or protein expression patterns alone does not directly address function, the knowledge of when and where a gene is expressed can provide valuable insights as to the potential role of a gene and has historically been instrumental in the discovery of developmentally regulated genes. For example, the earliest cDNA cloning experiments were based on the knowledge of tissue-specific expression and led to the isolation of cDNA clones for globin [39] and lysozyme [50]. Subsequent to the isolation of highly expressed genes such as globin, cDNA subtraction strategies were developed in order to reveal lower-abundance differentially expressed genes and led to the discovery of biologically important genes, such as the T cell receptor [29,63] and the myoD transcription regulator [18].

Recognition of the value of the examination of expression patterns led to the development of a plethora of more advanced technologies, such as cDNA microarrays [22], SAGE [58] and cDNA display [37] aimed at the simultaneous measurement of tens to several thousand genes in the target samples. However, a major restriction of most mRNA profiling approaches is the relatively large amount of starting mRNA required, thus limiting studies to the examination of pools of several million or at best several thousand cells. The ability to apply expression profiling to smaller samples including single cells would be beneficial for both basic research and clinical molecular diagnosis. However, since the total RNA content of mammalian cells is in the range 20–40 pg [46,54] and only 0.5–1.0 pg of this is mRNA, any attempt at single-cell profiling must be capable of dealing with a total of $10^5$–$10^6$ mRNA molecules. Despite this considerable limitation, over the last decade a multitude of amplification procedures have been developed in

Copyright © 2000 John Wiley & Sons, Ltd.

order to tackle mRNA expression profiling specifically at the level of a single mammalian cell. This review will focus on approaches that have been employed to multiply gene expression patterns either in small numbers of cells or, wherever possible, in actual isolated single cells. The first half of this review will summarize the technical aspects of methods developed for single cell analysis and the latter half of the review will describe the areas of biological research that have benefited from single-cell expression analysis.

### Technical approaches to single-cell profiling

In the late 1980s, Rapopolee and colleagues described a protocol known as 'single-cell mRNA phenotyping', which was developed for the analysis of multiple genes (10 or more) in small samples, including single cells [44,45]. In the original single-cell mRNA phenotyping method, following total RNA isolation, cDNA was prepared in a reverse transcriptase (RT) reaction using an oligo dT primer and separate gene-specific PCRs were carried out on aliquots of the total cDNA [45]. The method can detect as few as 100 mRNA molecules added to the RT reaction and was estimated to be able to detect three-fold differences in mRNA abundance [45]. Since the development of single-cell mRNA phenotyping there have been many technical advances, including the ability to analyse several genes simultaneously within the same PCR reaction (multiplex analysis). Multiplex analysis has been used to examine up to seven separate genes in single Purkinje neurons and Bergmann glial cells following patch-clamp recording of cells identified *in situ* [48]. Estimates of the relative abundance of target genes using multiplex approaches can be made by comparing the intensity of the target band to a constitutively expressed endogenous gene amplified in the same PCR reaction [42]. Alternatively, a known quantity of competitor cRNA can be added to the reverse transcription reaction to provide an absolute measure of the number of target mRNA molecules present in the analysed cell [4,66].

Although single-cell mRNA phenotyping or multiplex analysis has proved useful in examining expression in single cells, it is limited by the number of genes that can be analysed in each individual cell. In order to examine all, or at least the majority, of genes expressed in individual cells, a variety of global amplification protocols have been devised. One of the first successful global approaches applicable to single cells used RNA polymerase rather than Taq DNA polymerase to amplify target sequences [24,56]. In RNA polymerase-based amplification, known as aRNA, total cDNA is prepared using a specialized oligo d(T) primer incorporating the sequence of an RNA polymerase promoter, and approximately 1000 RNA copies of each cDNA molecule are generated in an *in vitro* RNA polymerase reaction [24,56]. When applied to single cells, the reverse transcriptase reaction is carried out directly on the cell contents and amplified aRNA is produced following cDNA purification and second strand synthesis [56]. Over the last decade aRNA has proved a successful and reliable method [32] and has been adapted for cDNA display [16,40] and recently has been used to generate probes for cDNA arrays from as little as 10 ng of total human RNA, equivalent to the RNA content of around 300 cells [59].

Around the same time that the aRNA amplification protocol was developed, two global RT–PCR methods were developed, both relying on utilizing the mRNA poly(A) tail for the first priming site, and creating a second priming site using the template independent polymerase terminal transferase [6,8]. These methods differ from one another primarily in that one approach (the Belyavsky method) focused on the amplification of full-length cDNA products [6], whereas the second method (PolyAPCR) was aimed at preserving the relative abundance of transcripts [8]. The major advantage in using the Belyavsky method is that, unlike PolyAPCR, both 5′ and 3′ sequences are amplified, thereby increasing the amount of sequence information available and allowing the detection of 5′ changes, such as differential promoter use or alternative splicing. However, in order to avoid selective amplification of shorter cDNA products, the Belyavsky method employs several purification steps [6], thereby introducing the potential for sample loss, thereby limiting its effectiveness in single-cell profiling. In contrast, biased amplification of cDNAs due to size is avoided in PolyAPCR by limiting the initial cDNA strand to around 100–700 bases, regardless of the size of the original RNA template, and amplification is achieved by the sequential addition of reagents to the starting

Copyright © 2000 John Wiley & Sons, Ltd. 

cell(s), thereby avoiding any losses associated with sample purification [8,10]. Although the simplicity and representative nature of PolyAPCR lends itself to multiple single-cell analysis [7,8,9,52,66] and quantitative studies [2,8,14,43,60], the 3′ nature of the PCR product (PolyAcDNA) makes it unsuitable for the analysis of changes in the 5′. Both amplification procedures have been used to prepare single-cell cDNA libraries [1,23] and have been adapted for cDNA subtraction [9,28,34,61] and cDNA display [12,30]. Due to the speediness and simplicity of the PolyAPCR method, it can be readily applied to hundreds of samples [66] and the resultant PolyAcDNA products have been widely used as probes in differential screening approaches. For example, PolyAcDNA probes have been prepared from micro-dissected mouse embryo tissues [57], antibody-fractionated human haematopoietic precursors [41], single murine haemopoietic precursors [15,61] and, more recently, PolyAcDNA probes from *Drosophila* follicle cells have been used for high density microarray screening [11].

A fourth global RT–PCR method (known as TPEA) has recently been described which uses the poly(A) tail for the first priming site and creates a second site by priming second strand cDNA synthesis with a primer consisting of both unique and degenerate sequences [20]. As with PolyAPCR, TPEA can be applied directly to the cell contents without RNA purification and the product is restricted to the 3′ end of each mRNA. So far TPEA has been used to detect expression of housekeeping genes and receptors in whole cells and fractions of cytoplasm sampled from individual cells following patch-clamp recording [20,21]. Finally, a recent report has described a method which combines aspects of aRNA and PolyAPCR, which in principle is able to generate representative full-length cDNA from single cells [64].

### Biological applications of single cell profiling

Although the technological improvements listed above are clearly important, the most important factor in single-cell expression profiling is the correct identification and isolation of the target cell. A variety of approaches have been developed for cell identification, based on morphology, cell location, presence of surface epitopes, physiological function and the behaviour of sibling cells. Here a selection of approaches will be outlined in order to illustrate the advantages of single-cell profiling.

Single-cell analysis is a potential important tool in the study of neoplasia, since tumour and leukaemic cells develop alongside their normal counterparts and are characterized by increasing cellular heterogeneity during the course of the disease. Analysis of individual tumour or leukaemic cells provides an elegant means of teasing out expression patterns in malignant cells free from contaminating cells and has the potential to further basic research and clinical pathology. For solid tumours, laser capture microdissection (LCM) offers one of the most promising means of isolating cells based on direct microscopic visualization of tissue sections [25]. Gene-expression profiles using gene-specific RT–PCR and micro-arrays have been obtained following LCM applied to human breast cancer sections [49] and LCM has been used to generate amplified aRNA probes for cDNA arrays from small numbers of rat neurones [38]. With recent improvements in both visualizing [26] and acquiring [51] single cells from pathological sections, it is likely that LCM will be a tool for single-cell profiling in tumours.

The ability to obtain single-cell dispersions readily and the presence of immunologically detectable cell-surface markers has greatly enhanced the identification and molecular profiling of leukaemic and lymphoma cells. For example, following microscopic isolation based on morphological and immunological staining, Trumper and colleagues applied PolyAPCR to individual Hodgkin's and Reed–Sternberg cells isolated from patients with Hodgkin's disease [52,53]. The combination of fluorescence-based cell sorting (FACS) and PolyAPCR has also been used to examine and compare expression patterns in normal and leukaemic haematopoietic subpopulations [33,47] and has led to the identification of a tumour-suppressor gene which is downregulated in pre-leukaemic disease [41]. Recent advances in tissue fixation, RNA recovery and FACS analysis [5] make it likely that cell fractionation methodologies similar to those developed for examining haemopoietic disorders will be increasingly used for the analysis of solid tumours.

In addition to aiding the study of tumours and leukaemia, single-cell profiling approaches have been particularly useful in examining haemopoiesis,

Copyright © 2000 John Wiley & Sons, Ltd.

early embryonic differentiation and cell physiology. As well as using antibody-based fractionation protocols to enrich for defined precursors [13,17,41,65,67], the ability to grow haemopoietic precursors in culture, has allowed the identification and molecular characterization of individual cells on the basis of their developmental capacity [9,14,66]. The general principle of this approach (known as sibling analysis; see [9] for details) is to grow individual precursors *in vitro* under non-restrictive growth conditions and allow them to divide two or five times to generate a 'colony start' of 4–32 cells. From each colony start, one or more cells are withdrawn for global amplification and the remaining sibling cells are grown separately to generate secondary colonies. The differentiation capacity of the lysed cell(s) used for RT–PCR is then inferred from the colony types arising from the living siblings. Since the culture conditions used result in synchronous differentiation [9,66], coupling single-cell profiling to functional developmental outcome in the form of sibling analysis provides a direct and precise examination of lineage-specific gene expression. Furthermore, since the haematopoietic precursors analysed by sibling analysis generally amount to less than 1% of starting haematopoietic tissues, it is unlikely that the expression patterns uncovered would be detected using expression methods applied to bulk populations. One frequently observed feature of single cells undergoing differentiation is the strikingly transient nature of expression patterns. For example, the imprinted tumour suppressor gene H19 is transiently expressed specifically at the onset haemopoietic lineage commitment and is low or undetectable at earlier or later stages of differentiation [41]. Similarly, examination of the expression of retinoic acid receptors (RAR) $\alpha$ and $\gamma$ in enriched populations and single cells revealed transient expression predominantly in cells destined to become granulocytes [35]. This observation led to the analysis of haematopoietic precursors in RAR $\alpha$ and $\gamma$ null animals and the discovery of a requirement for RAR $\alpha$ and $\gamma$ expression during terminal granulocytic maturation [35]. Recently, the scope of sibling analysis has been extended to the simultaneous assessment of genomic methylation patterns and mRNA expression in growing T cell clones [27].

Since oocytes, eggs and single-cell fertilized embryos represent the most readily recognizable and biologically important single cells in multicellular organisms, it is not surprising that single-cell profiling has been used to study early embryos [1,31,43]. Expression studies of oocytes, eggs and one-cell embryos is greatly helped by the fact that they are large and contain around 50–100-fold more mRNA than somatic cells ($1–3 \times 10^7$ mRNAs/cell; see discussion in [43]). Studies of early embryos have revealed frequent transient expression patterns similar to those seen in haematopoietic precursors, which are thought to reflect embryonic genome activation and the initiation of embryonic differentiation [19,36,43]. Latham and colleagues have applied quantitative RT–PCR to mRNA from embryos treated with transcription inhibitors and from enriched polysomes in order to evaluate gene-regulation due to alterations in adenylation of existing mRNAs or recruitment of mRNAs to the transcriptional machinery [43,60]. Their results indicate that a subset of G protein $\alpha$ subunits are regulated by *de novo* cytoplasmic adenylation of existing mRNAs at the 1–2 cell stage [43] and differential polysomal association of transcription factor and ribosomal protein mRNAs in metaphase II oocytes, 1 cell-stage embryos and 2 cell-stage embryos [60].

The third area of biological research that is frequently associated with single-cell mRNA profiling is that of cell physiology (reviewed in [21]). As with embryology and haematology, the application of single-cell mRNA profiling to cell physiology has been greatly facilitated by the prior development of a variety of methods for the identification, characterization and isolation of individual cells. For example, the experience gained in cell manipulation through patch-clamp analysis has facilitated the application of amplification protocols applied to cytoplasm extracted from single cells [21,24,56]. By combining physiological analysis and quantitative assessment of transcript levels in individual cells, it is possible to establish associations between mRNA expression physiological function [4]. Knowledge of the physiology and anatomy underlying olfactory sensory perception led to the isolation of a putative mammalian pheromone receptor from cDNA libraries constructed from single sensory neurons [23].

Single-cell expression profiling studies are enabling researchers to tackle the cellular complexity of higher eukaryotes. Single-cell studies of the type described in this review represent one of the few approaches suitable for the examination of rare,

Copyright © 2000 John Wiley & Sons, Ltd. 

biologically important cell types such as stem cells [3,55,62]. In addition to improving molecular analysis of limited amounts of clinical samples, single-cell profiling methodologies are also proving to be invaluable tools for uncovering novel patterns of gene expression linked to normal differentiation. Given the plethora of single-cell profiling approaches available, the choice of which method to use can be tailored to the requirements of each individual study. Factors to be considered when choosing the appropriate method include the number of samples to be processed, the need for quantitative analysis, the time required for sample preparation, whether full-length or short ESTs are required and the overall costs involved. Although single-cell approaches are currently clearly productive, in order to realize the true potential of these powerful techniques, more research will be required to establish the reliability and sensitivity of amplification and improved means of cell identification and isolation.

### Acknowledgements

I would like to thank the members of my laboratory for their valuable contributions to the development of some of the single-cell expression profiling approaches discussed. I am very grateful to Penny Johnson and Tania Nolan for reading the manuscript and the resultant helpful discussions and feedback. G.B. is currently an AstraZeneca senior research fellow.

### References

1. Adjaye J, Daniels R, Bolton V, Monk M. 1997. cDNA libraries from single human preimplantation embryos. *Genomics* **46**: 337–344.
2. Al-Taher A, Bashein A, Nolan T, Hollingsworth M, Brady G. Global cDNA amplification combined with real-time RT–PCR: accurate quantification of multiple human potassium channel genes at the single cell level. *Comp Funct Genomics; Yeast.* **17**: 201–210.
3. Bach S P , Renehan AG, Potten CS. 2000. Stem cells: the intestinal stem cell as a paradigm. *Carcinogenesis* **21**: 469–476.
4. Baro DJ, Levini RM, Kim MT, *et al.* 1997. Quantitative single-cell-reverse transcription–PCR demonstrates that A-current magnitude varies as a linear function of *shal* gene expression in identified stomatogastric neurons. *J Neurosci* **17**: 6597–6610.
5. Barrett MT, Glogovac J, Porter P, *et al.* 1999. High yields of RNA and DNA suitable for array analysis from cell sorter purified epithelial cell and tissue populations. *Nature Genet* **23**: 32–33.
6. Belyavsky A, Vinogradova T, Rajewsky K. 1989. PCR-based cDNA library construction: general cDNA libraries at the level of a few cells. *Nucleic Acids Res* **17**: 2919–2932 [published erratum appears in *Nucleic Acids Res* 1989 July 25; **17**(14):5883].
7. Berardi AC, Wang AL, Levine JD, Lopez P, Scadden DT. 1995. Functional isolation and characterization of human hematopoietic stem-cells. *Science* **267**: 104–108.
8. Brady G, Barbara M, Iscove NN. 1990. Representative *in vitro* cDNA amplification from individual hemopoietic cells and colonies. *Methods Mol Cell Biol* **2**: 17–25.
9. Brady G, Billia F, Knox J, *et al.* 1995. Analysis of gene-expression in a complex differentiation hierarchy by global amplification of cDNA from single cells. *Curr Biol* **5**: 909–922.
10. Brady G, Iscove NN. 1993. Construction of cDNA libraries from single cells. *Methods Enzymol* **225**: 611–623.
11. Bryant Z, Subrahmanyan L, Tworoger M, *et al.* 1999. Characterization of differentially expressed genes in purified *Drosophila* follicle cells: toward a general strategy for cell type-specific developmental analysis. *Proc Natl Acad Sci U S A* **96**: 5559–5564.
12. Candeliere GA, Rao Y, Floh A, Sandler SD, Aubin JE. 1999. cDNA fingerprinting of osteoprogenitor cells to isolate differentiation stage-specific genes. *Nucleic Acids Res* **27**: 1079–1083.
13. Chadwick BS, Brady G, Miller RG. 1993. Characterization of murine lymphokine-activated killer cell cultures separated according to cell size. *Cell Immunol* **146**: 1–10.
14. Cheng T, Shen HM, Giokas D, Gere J, Tenen DG, Scadden DT. 1996. Temporal mapping of gene expression levels during the differentiation of individual primary hematopoietic cells. *Proc Natl Acad Sci U S A* **93**: 13158–13163.
15. Claudio JO, Liew CC, Dempsey AA, *et al.* 1998. Identification of sequence-tagged transcripts differentially expressed within the human hematopoietic hierarchy. *Genomics* **50**: 44–52.
16. Crino PB, Trojanowski JQ, Dichter MA, Eberwine J. 1996. Embryonic neuronal markers in tuberous sclerosis: single-cell molecular pathology. *Proc Natl Acad Sci U S A* **93**: 14152–14157.
17. Cumano A, Paige CJ, Iscove NN, Brady G. 1992. Bipotential precursors of B cells and macrophages in murine fetal liver. *Nature* **356**: 612–615.
18. Davis RL, Weintraub H, Lassar AB. 1987. Expression of a single transfected cDNA converts fibroblasts to myoblasts. *Cell* **51**: 987–1000.
19. DeSousa PA, Watson AJ, Schultz RM. 1998. Transient expression of a translation initiation factor is conservatively associated with embryonic gene activation in murine and bovine embryos. *Biol Reprod* **59**: 969–977.
20. Dixon AK, Richardson PJ, Lee K, Carter NP, Freeman TC. 1998. Expression profiling of single cells using 3′ prime end amplification (TPEA) PCR. *Nucleic Acids Res* **26**: 4426–31.
21. Dixon AK, Richardson PJ, Pinnock RD, Lee K. 2000. Gene-expression analysis at the single-cell level. *TIPS* **21**: 65–70.
22. Duggan DJ, Bittner M, Chen YD, Meltzer P, Trent JM. 1999. Expression profiling using cDNA microarrays. *Nature Genet* **21**: 10–14.
23. Dulac C, Axel R. 1995. A novel family of genes encoding putative pheromone receptors in mammals. *Cell* **83**: 195–206.

Copyright © 2000 John Wiley & Sons, Ltd. 

24. Eberwine J , Yeh H , Miyashiro K, *et al*. 1992. Analysis of gene expression in single live neurons. *Proc Natl Acad Sci U S A* **89**: 3010–3014.
25. Emmert-Buck MR MR, Bonner RF, Smith PD, *et al*. 1996. Laser capture microdissection. *Science* **274**: 998–1001.
26. Fend F, Emmert-Buck MR, Chuaqui R, *et al*. 1999. Immuno-LCM: laser capture microdissection of immuno-stained frozen sections for mRNA analysis. *Am J Pathol* **154**: 61–66.
27. Fitzpatrick DR, Shirley KM, McDonald LE, *et al*. 1998. Distinct methylation of the interferon gamma (IFN-gamma) and interleukin 3 (IL-3) genes in newly activated primary CD8(+) T lymphocytes: regional IFN-gamma promoter demethylation and mRNA expression are heritable in CD44(high)CD8(+) T cells. *J Exp Med* **188**: 103–117.
28. Foot HCC, Brady G, Franklin FCH. 1996. Subtractive hybridisation. In *Plant Molecular Biology Laboratory Manual*, Clark M (ed.). Springer Verlag: Berlin.
29. Hedrick SM, Nielsen EA, Kavaler J, Cohen DI, Davis MM. 1984. Sequence relationships between putative T-cell receptor polypeptides and immunoglobulins. *Nature* **308**: 153–158.
30. Ivanova NB, Fesenko IV, Belyavskii AV. 1994. Novel method of comparative gene-expression analysis and identification of differentially expressed messenger-RNAs. *Mol Biol* **28**: 848–853.
31. Jurisicova A, Antenos M, Kapasi K, Meriano J, Casper RF. 1999. Variability in the expression of trophectodermal markers beta-human chorionic gonadotrophin, human leukocyte antigen-G and pregnancy specific beta-1 glycoprotein by the human blastocyst. *Hum Reprod* **14**: 1852–1858.
32. Kacharmina JE, Crino PB, Eberwine J. 1999. Preparation of cDNA from single cells and subcellular regions. *Methods Enzymol* **303**: 3–18.
33. Kawagoe H, Humphries RK, Blair A, Sutherland HJ, Hogge DE. 1999. Expression of HOX genes, HOX cofactors, and MLL in phenotypically and functionally defined subpopulations of leukemic and normal human hematopoietic cells. *Leukemia* **13**: 687–698.
34. Kim MG, Chen C, Flomerfelt FA, Germain RN, Schwartz RH. 1998. A subtractive PCR-based cDNA library made from fetal thymic stromal cells. *J Immunol Methods* **213**: 169–182.
35. Labrecque J, Allan D, Chambon P, Iscove NN, Lohnes D, Hoang T. 1998. Impaired granulocytic differentiation *in vitro* in hematopoietic cells lacking retinoic acid receptors alpha 1 and gamma. *Blood* **92**: 607–615.
36. Latham KE, Rambhatla L, Hayashizaki Y, Chapman VM. 1995. Stage-specific induction and regulation by genomic imprinting of the mouse u2afbp-rs gene during preimplantation development. *Dev Biol* **168**: 670–676.
37. Liang P, Pardee AB. 1992. Differential display of eukaryotic messenger RNA by means of the polymerase chain reaction. *Science* **257**: 967–971.
38. Luo L, Salunga RC, Guo H, *et al*. 1999. Gene expression profiles of laser-captured adjacent neuronal subtypes. *Nature Med* **5**: 117–122.
39. Maniatis T, Kee SG, Efstratiadis A, Kafatos FC. 1976. Amplification and characterization of a beta-globin gene synthesized *in vitro*. *Cell* **8**: 163–182.
40. Miyashiro K, Dichter M, Eberwine J. 1994. On the nature and differential distribution of messenger-RNAs in hippocampal neurites – implications for neuronal functioning. *Proc Natl Acad Sci U S A* **91**: 10800–10804.
41. Núnêz C, Bashein AM, Brunet C, *et al*. 2000. Expression of the imprinted tumour-suppressor gene H19 is tightly regulated during normal hematopoiesis and is reduced in hematopoietic precursors of patients with Polycythemia Vera. *J Pathol* **190**: 61–68.
42. Pernas-Alonso R, Morelli F, di Porzio U, Perrone-Capano C. 1999. Multiplex semi-quantitative reverse transcriptase-polymerase chain reaction of low abundance neuronal mRNAs. *Brain Res Brain Res Protoc* **4**: 395–406.
43. Rambhatla L, Patel B, Dhanasekaran N, Latham KE. 1995. Analysis of G protein alpha subunit mRNA abundance in preimplantation mouse embryos using a rapid, quantitative RT–PCR approach. *Mol Reprod Dev* **41**: 314–324.
44. Rappolee DA, Mark D, Banda MJ, Werb Z. 1988. Wound macrophages express TGF-alpha and other growth factors *in vivo*: analysis by mRNA phenotyping. *Science* **241**: 708–712.
45. Rappolee DA, Wang A, Mark D, Werb Z. 1989. Novel method for studying mRNA phenotypes in single or small numbers of cells. *J Cell Biochem* **39**: 71–81.
46. Roozemond RC. 1976. Ultramicrochemical determination of nucleic acids in individual cells using the Zeiss UMSP-I microspectrophotometer. Application to isolated rat hepatocytes of different ploidy classes. *Histochem J* **8**: 625–638.
47. Sauvageau G, Lansdorp PM, Eaves CJ, *et al*. 1994. Differential expression of homeobox genes in functionally distinct CD34(+) subpopulations of human bone-marrow cells. *Proc Natl Acad Sci U S A* **91**: 12223–12227.
48. Schmidt-Ott KM, Tuschick S, Kirchhoff F, *et al*. 1998. Single-cell characterization of endothelin system gene expression in the cerebellum *in situ*. *J Cardiovasc Pharmacol* **31**: 1S364–366.
49. Sgroi DC, Teng S, Robinson G, *et al*. 1999. *In vivo* gene expression profile analysis of human breast cancer progression. *Cancer Res* **59**: 5656–5661.
50. Sippel AE, Land H, Lindenmaier W, *et al*. 1978. Cloning of chicken lysozyme structural gene sequences synthesized *in vitro*. *Nucleic Acids Res* **5**: 3275–3294.
51. Suarez-Quian CA, Goldstein SR, Pohida T, *et al*. 1999. Laser capture microdissection of single cells from complex tissues. *Biotechniques* **26**: 328–335.
52. Trumper LH, Brady G, Bagg A, *et al*. 1993. Single-cell analysis of Hodgkin and Reed–Sternberg cells: molecular heterogeneity of gene expression and p53 mutations. *Blood* **81**: 3097–3115.
53. Trumper LH, Brady G, Vicini S, Cossman J, Mak TW. 1992. Gene expression in single Reed–Sternberg cells of Hodgkin's disease: results from PCR-generated single-cell cDNA libraries. *Ann Oncol* **3**(suppl 4): 25–26.
54. Uemura E. 1980. Age-related changes in neuronal RNA content in rhesus monkeys (*Macaca mulatta*). *Brain Res Bull* **5**: 117–119.
55. van der Kooy D, Weiss S. 2000. Why stem cells? *Science* **287**: 1439–1441.
56. Van Gelder RN, von Zastrow ME, Yool A, *et al*. 1990. Amplified RNA synthesized from limited quantities of heterogeneous cDNA. *Proc Natl Acad Sci U S A* **87**: 1663–1667.
57. Varmuza S, Tate P. 1992. Isolation of epiblast-specific cDNA

Copyright © 2000 John Wiley & Sons, Ltd. 

clones by differential hybridization with polymerase chain reaction-amplified probes derived from single embryos. *Mol Reprod Dev* **32**: 339–348.
58. Velculescu VE, Zhang L, Vogelstein B, Kinzler KW. 1995. Serial analysis of gene expression. *Science* **270**: 484–487.
59. Wang E, Miller LD, Ohnmacht GA, Liu ET, Marincola FM. 2000. High-fidelity mRNA amplification for gene profiling. *Nature Biotechnol* **18**: 457–459.
60. Wang QX, Latham KE. 2000. Translation of maternal messenger ribonucleic acids encoding transcription factors during genome activation in early mouse embryos. *Biol Reprod* **62**: 969–978.
61. Weaver DL, Núñez C, Brunet C, Bostock V, Brady G. 1999. Single-cell RT–PCR cDNA subtraction. In *Molecular Embryology: Methods and Protocols*, Sharpe P, Mason I (eds). Humana: Totowa, New Jersey, 603–609.
62. Weissman IL. 2000. Translating stem and progenitor cell biology to the clinic: barriers and opportunities. *Science* **287**: 1442–1446.
63. Yanagi Y, Yoshikai Y, Leggett K, *et al*. 1984. A human T cell-specific cDNA clone encodes a protein having extensive homology to immunoglobulin chains. *Nature* **308**: 145–149.
64. Ying SY, Lui HM, Lin SL, Chuong CM. 1999. Generation of full-length cDNA library from single human prostate cancer cells. *Biotechniques* **27**: 410–412.
65. Ziegler BL, Lamping CP, Thoma SJ, Fliedner TM. 1995. Analysis of gene-expression in small numbers of purified hematopoietic progenitor cells by RT–PCR. *Stem Cells* **13**: 106–116.
66. Ziegler BL, Muller R, Valtieri M, *et al*. 1999. Unicellular-unilineage erythropoietic cultures: molecular analysis of regulatory gene expression at sibling cell level. *Blood* **93**: 3355–3368.
67. Zinovyeva MV, Zijlmans J, Fibbe WE, Visser JWM, Belyavsky AV. 2000. Analysis of gene expression in subpopulations of murine hematopoietic stem and progenitor cells. *Exp Hematol* **28**: 318–334.

The Genomics Website at Wiley www.wiley.co.uk/genomics

The **Genomics** website at Wiley is a new and **DYNAMIC** resource for the genomics community, offering **FREE** special feature articles and new information **EACH MONTH**.

Find out more about *Comparative and Functional Genomics*, and how to view all articles published this year **FREE OF CHARGE**!

Visit the **Library** for hot books in Genomics, Bioinformatics, Molecular Genetics and more.

Click on **Primary Research** for information on all our up-to-the minute journals, including: *Genesis*, *Bioessays, Gene Function and Disease*, and the *Journal of Gene Medicine*.

Let the **Genomics** website at Wiley be your **guide** to genomics-related web sites, manufacturers and suppliers, and a calendar of conferences.

WILEY

1516

Copyright © 2000 John Wiley & Sons, Ltd. 
